# Scalp Incisions With Stairstep Pericranial Edges to Minimize Sequalae from Poor Wound Healing in Supratentorial Brain Tumor Surgery: A Technical Note With Early Results

**DOI:** 10.1227/neuprac.0000000000000052

**Published:** 2023-09-22

**Authors:** Nicholas Popp, Ishan Singhal, Brandon Laing, Kate B. Krucoff, Max O. Krucoff

**Affiliations:** * Medical College of Wisconsin, Milwaukee, Wisconsin, USA;; ‡ Department of Neurosurgery, Medical College of Wisconsin, Milwaukee, Wisconsin, USA;; § Department of Plastic and Reconstructive Surgery, Medical College of Wisconsin, Milwaukee, Wisconsin, USA;; ‖ Department of Biomedical Engineering, Medical College of Wisconsin & Marquette University, Milwaukee, Wisconsin, USA

**Keywords:** Cerebrospinal fluid leak, Craniotomy, Resection, Surgical site infection, Surgical technique, Wound, healing

## Abstract

**BACKGROUND AND OBJECTIVES:**

Wound healing problems are especially prevalent in craniotomies for intra-axial brain tumors as patients often require radiation, chemotherapy, and chronic steroids. Although newer techniques such as minimally invasive approaches and routine vancomycin powder use have helped overall complication rates, poor skin healing remains a frustratingly persistent cause of morbidity. Therefore, here we describe the novel technique of elevating and closing a stairstep pericranial edge offset from the skin incision to protect hardware and support wound healing, and we report early outcomes using this technique.

**METHODS:**

Ninety-one consecutive patients underwent supratentorial, intra-axial brain tumor surgery with a single surgeon at a single institution using this technique. Patient demographics, pathology, adjuvant interventions, and other independent risk factors were analyzed.

**RESULTS:**

No wound-related complications requiring readmission, intravenous antibiotics, or reoperation were encountered at a median 3-month follow-up. There were also no surgical site infections, dehiscences, or cerebrospinal fluid leaks. Fifty-one patients (57.3%) had postoperative radiotherapy, 85 patients (93.4%) had perioperative steroids, and 56 patients (61.5%) had postoperative chemotherapy. Six patients (6.5%) were placed on a short course of oral antibiotics perioperatively due to concerns with initial scalp healing (ie, excessive scabbing at follow-up), none of whom progressed to infection or required further intervention. These are the cases where this technique is felt to have been most helpful by potentially preventing worse sequelae. One patient developed a shunt infection during this interval that required removal unrelated to the craniotomy site.

**CONCLUSION:**

Here we outline in detail the principles, design, and execution of incisions and closures with stairstep pericranial edges in supratentorial brain surgery. This technique was designed in consultation with plastic surgeons to provide an intact, vascularized layer of pericranium beneath the healing skin and over the bone graft/hardware to optimize wound healing conditions and prevent morbid sequelae in inevitable cases of poor initial healing. Early results are promising.

**ABBREVIATION:**
SSI: surgical site infection.

Wound healing problems are especially prevalent in patients undergoing craniotomies for intra-axial brain tumors given the nearly ubiquitous need for perioperative steroids, radiation, and chemotherapy, all of which inhibit skin healing.^1-3^ Furthermore, craniotomies require removing and replacing a bone graft with hardware, introducing foreign bodies that can become sources of persistent infection in cases of poor scalp healing. Recent tumor resection case series report culture-positive surgical site infection (SSI) rates between 2.0% and 4.4% using National Healthcare Safety Network SSI surveillance criteria.^4,5^ Wound dehiscence has been reported to be as high as 7.8% in other series.^6^

Because of potentially grave sequelae from poor wound healing (e.g., loss of bone flap, cerebral abscess, meningitis, etc.), there have been many efforts to reduce the risk of wound complications. For example, the use of vancomycin powder has become more routine in craniotomies and has been shown to reduce the incidence of SSI from 5% to 0.8% in 1 series.^7-10^ Although minimally invasive approaches to brain tumor resection have been described using the keyhole principles, current data do not support a difference in infection rates compared with traditional approaches.^11^ Thus, poor wound healing remains an important and persistent contributor to morbidity in neurosurgical patients.^12^

Because of these challenges, we collaborated with our plastic surgery colleagues (who are often asked to help address complications in the most difficult cases) to modify our approach to opening and closing the scalp to minimize the impact of suboptimal skin conditions on overall wound healing. Specifically, we began designing incisions offset from the bone edge and created a stairstep pericranial edge to provide a fully vascularized and intact tissue layer beneath the healing skin on closure. In this manner, all hardware and devascularized bone is protected by a vascularized tissue layer to aid in secondary intention in case of poor initial wound healing.^13^ In addition, the stairstep pericranium may minimize the direct cerebrospinal fluid (CSF) pressure on the incision, thus reducing the impact of pseudomeningoceles on scalp healing.

## Specific Aims

Here, we describe this technique and present our initial results as a series of 91 consecutive first-time intra-axial supratentorial brain tumor craniotomies without any wound complications requiring readmission or reoperation. To the best of our knowledge, this is the first time this stairstep pericranial edge technique has been described for supratentorial brain tumor surgery.

## METHODS

### Inclusion and Exclusion Criteria

Data were collected prospectively from consecutive cases between 10/14/2020 to 9/5/2022 and reviewed retrospectively in compliance with IRB protocol PRO00030059, resulting in a total of 91 patients. All patients underwent standard informed consent before surgery and had a primary supratentorial resection of intra-axial lesions using the stairstep pericranial edge. The participants and any identifiable individuals were consented to publication of his/her image before surgeries, consistent with another IRB-approved protocol PRO00030059. Multiple procedures performed on a single patient were only counted separately if they independently met inclusion criteria, were performed with a new incision, and included the pericranial edge. Infratentorial tumor resections, traumas, shunts, and secondary surgeries through previous incisions were not included. No meningiomas were included in this series because these cases are less likely to receive radiation.

### Patient Demographics

Patient demographics were obtained through chart review. Preoperative radiation was defined as any radiation treatment including the scalp ever before the procedure. Postoperative radiation was defined as any radiation treatment within 2 months after the procedure. Perioperative steroids were defined as any use of corticosteroids within 1 week before or 2 weeks after the procedure. Chemotherapy use was defined as any antineoplastic medication including biologics used within 3 months after surgery. Immunotherapy was defined as any immunotherapy used within 3 months leading up to or after surgery. Last follow-up was defined as the last time the incision was examined during a follow-up appointment with neurosurgery or neuro-oncology. Smoking status was defined as a current smoker at the time of surgery. Diabetes was defined as any patient having a diagnosis of Type I or II diabetes regardless of the level of glucose control. All patients received preoperative IV antibiotics within an hour before incision as part of routine care. Nonroutine antibiotic use was defined as any antibiotics prescribed at clinic follow-up in response to findings of excessive scabbing, skin irritation from glasses or hats, or other signs of suboptimal initial wound healing without evidence of infection.

### Exposure

#### Step 1—Designing an Offset Incision

**FIGURE 1.**
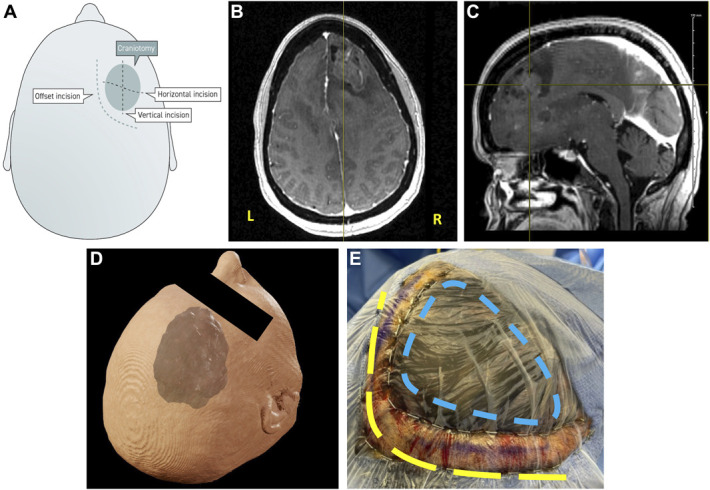
Offset incision design. **A**, Potential incisions for a given craniotomy. **B**, Axial and **C**, sagittal views of brain MRI used in surgical planning of a representative patient with a right frontal tumor. **D**, 3-dimensional rendering of patient's tumor, including nonenhancing tumor elements. **E**, Approach to surgical planning, showing the approximate needed craniotomy size (blue dashed line), offset scalp incision (purple line), and approximate stairstep pericranial incision (yellow dashed line).

The incision is planned with the help of neuronavigation, initially drawing out the intended size of the corticetomy and working outward from there. Each layer is given some offset to allow for appropriate exposure and easy closure (in contrast to many keyhole techniques). The skin incision is designed to be offset from the hardware, and it is generally planned to maintain major feeding arteries (Figure 1A-1D). Because all incisions are designed behind the hairline with minimal hair shaving, there is ample opportunity for an excellent cosmetic result even with a slightly longer incision (Figure 1E).

#### Step 2—Stairstep Pericranial Edge

**FIGURE 2.**
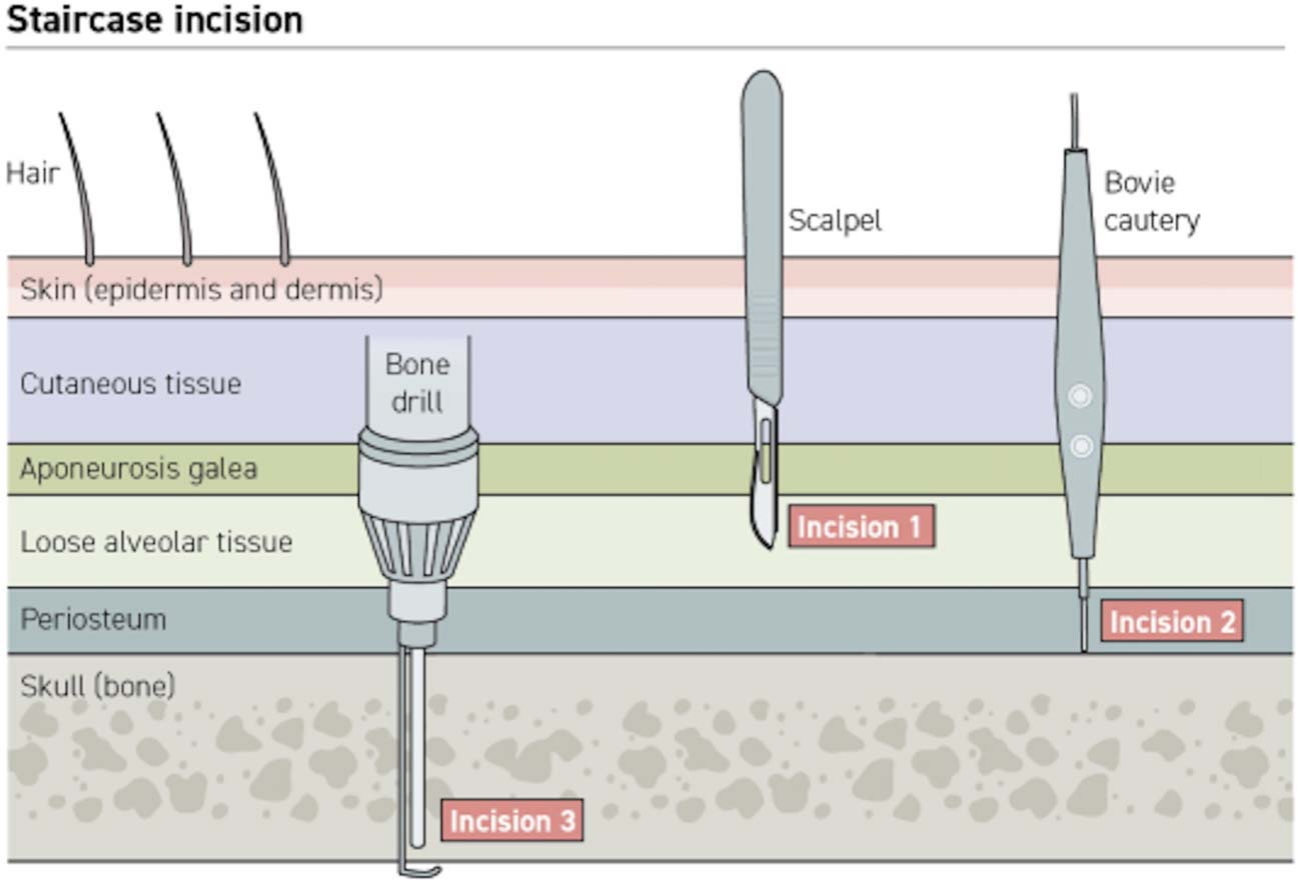
Stairstep pericranial incision concept. Schematic of layers of the scalp depicting offset incisions.

**FIGURE 3.**
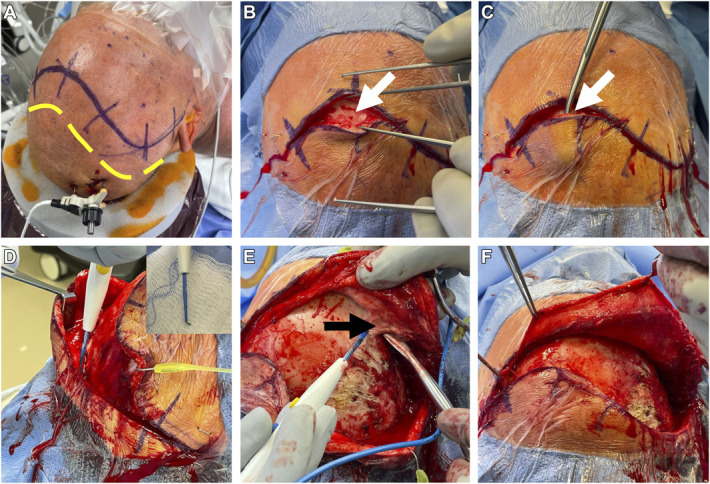
Example of an incision with a stairstep pericranial edge and contiguous temporalis muscle elevation. **A**, Planned incision (purple line) and corresponding planned pericranial incision (yellow dashed line). **B**, Incision through skin, cutaneous tissue, and galea, exposing native periosteum (white arrow). **C**, Elevation of galea with scalp away from periosteum (subgaleal and supraperiosteal), exposing the loose alveolar tissue to be dissected. **D**, Pericranial incision created with the bent tip monopolar (viewed from patient's side ride). **E**, Temporalis fascia attachment is separated from the skull at the superior temporal line (black arrow), allowing the muscle to be elevated in 1 piece with the pericranial edge and scalp. **F**, The stair-stepped pericranial edge elevated with the scalp edge and contiguous temporalis muscle.

To provide additional support to the healing scalp, a vascularized pericranial edge is raised in a stairstep manner, slightly offset from the scalp incision line (Figure 2). After the initial incision is made through the skin and galea, the galea of the surrounding scalp is then elevated off the periosteal layer and the alveolar layer is cut (Figure 3A-3C). Next, a monopolar cautery is used to incise the periosteum beyond the outer scalp incision down to bone (Figure 3D).

In cases involving the temporalis muscle (ie, pterional exposures), the muscle layer is kept in continuity with the periosteal layer and raised with the pericranial edge in 1 piece (Figure 3E). It should be noted that dissection in the subgaleal, supraperiosteal plane is safe in virtually all scalp locations except superficial to the anterior portion of the temporalis muscle over the keyhole just posterior to the lateral canthus, as this is where the frontalis branch of the facial nerve transitions layers and can be injured.^14^ It is important in these cases to always make sure that the stairstep is performed away from this area or not at all.

The pericranium is then separated from the bone using a periosteal elevator, being careful to elevate the white layer on the underside of the periosteum. This stair-stepped pericranial “lip” is then raised with the scalp in 1 piece (Figure 3F).

### Closure

#### Step 1—Stairstep Pericranial Closure

**FIGURE 4.**
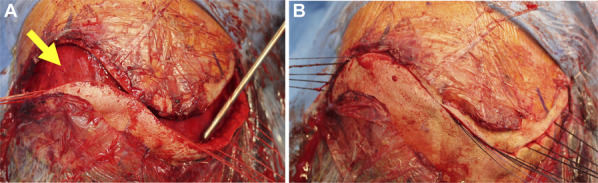
Stairstep pericranial closure. **A**, Pericranium is closed to either the residual pericranial edge or its overlying galea offset from the skin incision line using 2-0 CT1 Vicryl interrupted sutures. Note the complete coverage of bone and hardware with this vascularized layer before galeal closure (yellow arrow). **B**, Galeal layer is closed using 2-0 SH Vicryl inverted sutures in the standard fashion. Skin layer is closed using running nylon sutures.

**FIGURE 5.**
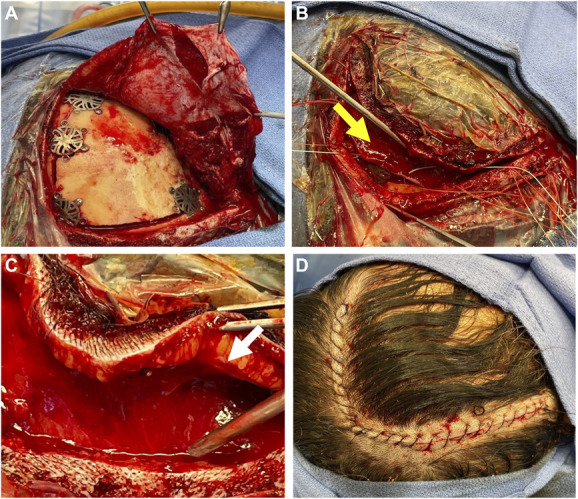
Second example of stairstep pericranial closure. **A**, Note that even with the offset incision, some of the hardware still lies directly below the incision, as intraoperative adjustments are sometimes made. **B**, Pericranial edge (yellow arrow) is closed to the residual pericranial edge or its overlying galea using 2-0 CT1 Vicryl single interrupted sutures. This covers the hardware completely even before galeal closure. **C**, Galeal layer (white arrow) is then closed in a separate layer using 2-0 SH Vicryl inverted sutures. **D**, Skin layer is closed using running nylon sutures.

After the dura is closed and the bone is replaced, the pericranial edge is closed with 2-0 CT1 Vicryl (Ethicon Inc.) single interrupted sutures that reach from the edge of the cut pericranium to a deep, offset portion of the galea underlying the surrounding scalp, or to the cut edge of the pericranium if accessible. The closure is not generally water-tight, but it is stretched to ensure that all hardware is covered and that the suture line is supported from below with this layer. This constitutes the stairstep closure (Figures 4A and 4B and 5A-5D, Supplemental Video).

**VIDEO.** Step-by-step demonstration of an offset scalp incision with a stairstep pericranial edge in a supratentorial brain tumor resection.

#### Step 2—Galea and Scalp

The galea is then closed with 2-0 Vicryl (Ethicon Inc.) inverted sutures in the common fashion, followed by a running 3-0 or 4-0 nylon in most cases.

## RESULTS

Table presents the demographic information from our patients. There were no wound-related complications that required readmission or surgical intervention in this series, such as infections, dehiscence, or CSF leakage across a median 3-month follow-up period. There was 1 case of a shunt infection unrelated to the primary surgical incision that required removal and external ventricular drain placement. Any patient who had excessive scabbing or concern for skin irritation at the incision line at the first clinic follow-up (ie, at the site of eyeglasses passing over the ear) was started on a short course (10-14 days) of prophylactic oral antibiotics (cephalexin 500 mg four times a day as the first line). There were 6 patients (6.6%) who met these criteria (Table), and none of these cases developed purulent drainage, fevers, systemic symptoms, spreading erythema, or other stigmata of infection. These cases are where the offset pericranial layer was likely the most helpful in preventing further sequalae.

**TABLE.** Demographic and Treatment Course Data

Demographic information	Percentage of patients
Age	Average 55.5 ± 16.4 y SD
Male:female ratio	46:45
Prior cranial surgery	12.1% (n = 11)
Diagnoses	
Cyst or other benign intra-axial pathology	15.4% (n = 14)
Low-grade glioma	22.4% (n = 20)
High-grade glioma	38.5% (n = 35)
Metastasis	24.2% (n = 22)
Intervention	
Preoperative radiation	7.7% (n = 7)
Postoperative radiation	57.3% (n = 51)
Perioperative steroids	95.5% (n = 87)
Chemotherapy	62.9% (n = 56)
Immunotherapy	14.3% (n = 13)
Smoking status	12.4% (n = 10) current smokers
Diabetes	
Type I	3.3% (n = 3)
Type II	6.6% (n = 6)
Perioperative steroid use	95.5% (n = 85)
Median length of hospital stay	5 d
Follow-up	3.5 mo (median); 2.8 mo (mean)
Postoperative antibiotic use	6.6% (n = 6)

## DISCUSSION

For neurosurgeons familiar with treating patients with malignant tumors, poor skin healing is a matter of “when,” not “if.” Therefore, it is important to minimize the clinical impact and potentially morbid sequelae when this occurs. It is this principle that underlies the design of the offset incision and pericranial edge described in this series. Data presented here show 91 consecutive cases without wound-related reoperations or readmissions despite frequent use of steroids and perioperative chemo-radiation.

**FIGURE 6.**
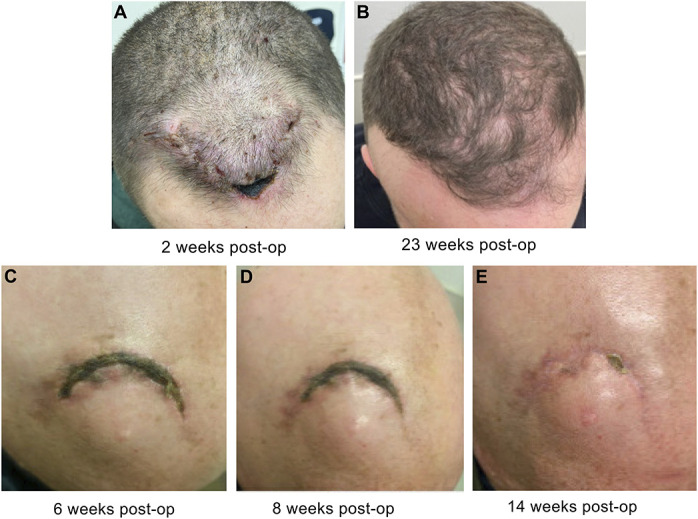
Examples of initial poor wound healing that healed with time and without surgical intervention, likely supported by the underlying intact pericranium. Both patients were placed on prophylactic short-term oral antibiotics. **A**, Postoperative pictures at 2 weeks and **B**, 23 weeks of a patient with poor healing at the skin edges due to both preoperative and postoperative radiation. The wound subsequently healed without intervention. **C**, The patient with multiple intracranial metastases who required placement of an Ommaya reservoir and urgent cranial radiation 72 hours after surgery (note: the patient was not included in this series as there was no tumor resection). An offset incision and stair-stepped pericranial edge were used. Despite the poor healing at the wound edges and implanted hardware, she healed on her own over time with the progress shown from 6 weeks, **D**, 8 weeks, and **E**, 14 weeks postoperation.

Two examples of poor wound healing in our series that may have led to more serious complications in other circumstances are presented in Figure 6. Figure 6A and 6B presents a young man who had received prior cranial radiation and additional postoperative radiation after his surgery for a cingulate gyrus glioblastoma. He presented to clinic 2 weeks postoperatively with extensive scabbing and a poorly healing wound. He was placed on short-term oral antibiotics, and, fortunately, his wound healed without further intervention. With other techniques, the concern for seeding underlying bone and hardware through the scab would have been high, whereas the underlying intact pericranial layer here likely supported its spontaneous healing. Similarly, the case shown in Figure 6C-6E represents a woman with widely metastatic intracranial disease who required an Ommaya reservoir placement and emergent whole-brain radiation 72 hours after surgery. Although this patient was not included in this series because it was not a brain tumor resection, it is shown as a demonstration of principle as an offset incision with a pericranial edge was used here as well. She was also placed on short-term prophylactic antibiotics and proceeded to heal without any further intervention because her hardware was never seeded. Another case in this series (not pictured) included a patient who broke a running nylon suture on postoperative day 5 while combing his hair, yet he also healed on his own without intervention. Such suboptimal situations like these are unavoidable in real-world practice, providing inspiration for the technique outlined here.

We note that these are early results and a larger series with longer follow-up is necessary to determine the true complication rate, which we plan to provide when it becomes available. However, using the National Healthcare Safety Network SSI surveillance criteria standards,^15^ our early results compare favorably to the literature. For example, when we compare our results with an expected 4.4% wound complication rate reported by similar case series^7^ using a χ^2^ 2-tailed test (which allows a priori for the possibility of better or worse outcomes), we find a *P* value of 0.04, suggesting a statistically significant difference. However, when compared with a 2% SSI rate reported by other cases series, the same test fails to reach statistical significance (0.1527), suggesting it is either no different or underpowered.^6^ We again note that while 6 patients in our series were given prophylactic oral antibiotics at the clinic follow-up due to poor initial wound healing (ie, excessive scabbing or irritation from glasses/hats), none of these individuals met the National Healthcare Safety Network SSI surveillance criteria standards for SSI^15^ nor did they show any clinical signs or symptoms of infection.^15^ Although the trend in modern neurosurgery has been toward making smaller, linear incisions, here we advocate making offset incisions when behind the hairline. Although linear incisions have the advantages of efficiency, simplicity, and consistency, they also have disadvantages, namely (1) the incision is made directly over the bone graft, (2) retraction must be maintained on the skin edges throughout the case, and (3) any CSF pulsations may put the wound under direct tension. For surgeons who still prefer to use linear incisions, it is possible that adapting a stairstep pericranial layer might provide a similar benefit. This is yet to be tested. Theoretical disadvantages to the offset incision with a stairstep pericranial edge include (1) increased length of surgical exposure and closure, (2) increased subgaleal dissection and deadspace, and (3) increased blood loss. In this series, the qualitative impact of these concerns was not appreciable.

### Limitations

The intent of this article is to describe the technical aspects of and theory behind a novel technique and to report early results. We emphasize that these are early, descriptive results without a control group, and we anticipate following up with more long-term data when it becomes available. This technique is not expected to eliminate all intracranial infections because it does not directly prevent or mitigate intraoperative contaminations or hematogenously spread bacteria. We anticipate infections from these sources (ie, deeper infections) showing up in our data with more time. We also note that this work lacks a control group and does not directly compare this method against any other, and there are many factors that contribute to wound healing. All literature comparisons are made as a general reference only, and no conclusions about the specific contributions of this technique to the lower SSI rate should be made. This series represents a single surgeon's experience, so extrapolation should be used with caution, and there may be a learning curve to adopting this technique.

## CONCLUSION

Here we describe the technique of creating offset incisions with stairstep pericranial edges for use in supratentorial brain surgery (Video). This technique provides an intact, vascularized layer of pericranium beneath the healing incision and over the bone graft/hardware to optimize wound healing conditions and minimize morbid sequelae when skin initially heals poorly. This study serves as a technical description of how to perform this exposure and closure, as well as an examination of the principles behind its design and a report of early outcomes. Future prospective, controlled studies will be needed to assess whether this technique actually affects surgical complication rates. Early results are promising.
